# Dental Abstracts

**Published:** 1854-10

**Authors:** Geo. Watt


### Dental Abstracts.

Owing to pressure of other matter, we have gotten far behind in this department of our publication. Indeed the various Dental periodicals are now so well filled with interesting and useful matter, that it is impossible to even notice all which is published. In our last we noticed several articles on the use of crystal gold, and to give the Profession as much light as possible on the use of this new article, we omitted noticing several articles which we shall now refer to, and give some abstracts from.

In the April number of the News Letter, we have first a valedictory address to the graduating class of the Philadelphia College of Dental Surgery, by Prof. E. Townsend.

The Doctor after having spoken of the nature and duties of the Dental Practitioner—the faithful manner in which the graduates had discharged there duties as students, &c., discourses as follows of our art as a science :

Dentistry is usually spoken of as a branch of the great healing art, but, in point of fact, it has grown, not out of the stem, but up from the root of the tree of remedial science, and as it has not sprung from, so it does not depend upon the older trunk, but stands beside it, deriving its separate nutrition from the same soil indeed, yet by the independent energies of its own vitality. Hitherto, in fact, it has been indebted rather for shadow than sunshine to the elder-born growth. A thrifty sapling it has proved, with roots and branches of its own; distinct in its vital economy, though kindred in origin; distinct, also, in its fortunes, and necessarily so in the conditions and policy of its culture. Moreover, it has already so far matured, that it is full time for it to be set out by itself for larger room to grow and ripen its proper fruits.

Doctor of Dental Surgery is a comparatively new patent of nobility in the heraldry of science, and necessarily institutes the relations and duties of a new order in the diplomatic ranks. To this service we have pledged a generous devotion. We have enlisted in the regular army of advance, and the consciousness that its fortunes must be vitally affected by our conduct in the field, cannot fail to fire the zeal, and steady the fidelity due to the cause.

What does it ask at our hands; and how shall we best answer its great demands?

Very rapidly and successfully, yet still very recently, the profession has advanced from the sheer chaos of impiricism to the form and order of a regularly systematic art; so founded upon principles, and so justified by experience, as entitles it to the character of an integral science. It has also richly provided itself with the apparatus and method of future growth and progressive achievment. Already we are in possession of elementary treatises in every department of the study; we have an able periodical literature, and colleges for thorough and and comprehensive education are springing up with a rapidity and a capability almost equal to the demands of the times. We have so well advanced in the transition stage of our progress, if not quite passed it, that the elements of a permanent order are rapidly arraying themselves into the most efficient forms.

### CLAIMS OF PERIODICAL PUBLICATIONS.

In relation to the claims of periodical publications and the general interests of the profession, the Doctor takes high and we think just ground. In this connection the oath exacted by Hippocrates is given, which shows a zeal for the Profession and its teachers.

The standard and periodical publications, devoted to our art, have unquestionable claims upon your support. Every dentist, worthy of the name, should consider himself an agent for their circulation, and a contributor, by implied contract, to their stores of information. Every liberal profession, as much as that of religion, has, besides its sanctity to be guarded, its interests and usefulness to be promoted. The lawyer the physician, the naturalist, the dentist, is a sort of priest of his order, and owes to it the required fidelity, sacrifice and service. The philosophers of Greece exacted a sacramental vow from the disciples whom they initiated into the mysteries of their Schools. Hippocrates administered an oath to the adepts of the healing art. I will read it to you, both for the curiosity and the instructive suggestions it contains:

"I swear by Apollo, the Physician, by Æsculapius, by Hygiea, by Panacea, and all the gods and goddesses, calling them to witness, that I will fulfil religiously, according to the best of my power and judgment, the solemn promise, and the written bond which I now do make: I will honor as my parents the master who has taught me this art, and endeavor to minister to all his necessities: I will consider his children as my brothers, and will teach them ny profession, should they express a desire to follow it, without remuneration or written bond. I will admit to my lessons, my discourses, and all my other methods of teaching, my own sons, and those of my tutors, and those who have been inscribed as pupils and have taken the medical oath, and *no one* else. I will prescribe such a course of medicine as may be best suited to the constitution of my patients, according to the best of my power and judgment, seeking to preserve them from anything that might prove injurious. No inducement shall ever lead me to administer poison, nor will I be the author of such advice. I will maintain religiously the purity and integrity, both of my conduct and my art. Into whatever dwellings I may go, I will enter them with the sole view of succouring the sick. If, during my attendance, or even unprofessionally in common life, I happen to hear of any circumstances which should not be revealed, I will consider them a profound secret, and observe on the subject a religious silence. May I, if I religiously observe this my oath, and do not break it, enjoy good success in life, and in the practice of my art, and obtain general esteem forever. Should I transgress and become a perjurer, may the reverse be my lot."

Now, whatever the altered circumstances of the times have made obsolete and inapplicable in the grand summary of professional obligations, the principles which it recognizes are of perpetual obligation. None of them could be better presented, and some of them I might not have chosen to express; but there is a parity of conditions which will not fail to warrant their application to ourselves, our relation to each other, to our calling, to our patients and to the public. But especially are the sanctity and the devotedness of the order to which these principles of conduct, and these sentiments of fraternity apply, as well in our cases as in any other, well worthy of acceptance and observance. Our young profession demands of us equal ardor of service, and equal jealousy of defense, as medicine did in the distant age of its early infancy: above all things, it needs the spirit and corporate enthusiasm, and priestly purity, and sacredness of dedication that correspond to its divine origin and beneficent aims. The idea that I would enforce here must be obvious enough, and sufficiently warranted by its practical results, but I am tempted to strike the thought still deeper to the grand principle upon which it rests.

In connection with the above, and as the close of this very appropriate address we give another extract.

A man is to be measured by his merits, notwithstanding that a diploma is *prima facia* evidence, and a worthy distinction, of character and standing. Still it is your duty to repress and discredit unfounded pretension by all the means fairly and effectually in your power. This, in general, will be best accomplished by fully and decidedly answering to every claim of the accomplished and regular professors of our art, and by as decidedly refusing to admit those of the unworthy and incapable. A great advantage—an indispensable one—of the corporate organization which we have already urged is, that its honorable reciprocities withheld may act as distinctions and penalties upon groundless pretension. Just as the disciples of Hippocrates were sworn to admit to the fraternity, "those who had been inscribed as pupils, had taken the medical oath and no one else," so we are bound to refuse fraternity to the irregulars, who repudiate the essential obligations of the profession, and discredit its name. We do not expect, and I do not think we desire quack laws of the legislature to repress abuses, but we require quack tests established by ourselves, and well received by the community, by which they may be speedily and certainly extirpated. This is our proper duty, we must address ourselves to it, and the means within our command are, in general terms, the improvement of the system of private tutorship, the active support of the collegiate system, now fast rising into confidence among us; the liberal encouragement of our periodical publications, the organization of efficient dental associations among practitioners for their mutual improvement and protection, in every district where such parliaments of progress are practicable, and also the decided establishment of all those distinctions which serve to certify character and standing among ourselves, and instruct the public judgment in deciding upon professional pretensions.

These things, and all which they include, we would press upon your consideration and commend to your hearty observance. The profession, to adopt the battle orders of Lord Nelson, "expects every man to do his duty" To you is assigned the post of honor, and we will not allow ourselves to doubt your worthiness of the trust, or your fidelity and efficiency in performing it.

There is a moral chivalry, nobler in tone, pitch and purpose, because more beneficent, than that of arms. Are you baptized with its spirit, capable of its service, devoted to its achievements? Then you will exert its energies, and secure and enjoy its victories.

I began by bidding you welcome to your professional honors. I close by committing you to the divine care in your public duties and personal destiny.—FAREWELL.

### NEW NON-CONDUCTING SUBSTANCE FOR CAPPING NERVES.

In the April number of News Letter, we have an article on non-conducting substances for capping nerves, by C. A. DuBouchet, M.D., D.D.S. After testing many articles used by the profession, the Dr. has recommended horn. He regards its indestructibility and similarity and affinity of structure to the teeth itself, as qualities admirably adapting it for this purpose.

The first material used for capping nerves was sheet gold filed proper shape, so as to be introduced and retained in the cavity. Sheet lead was afterwards substituted, in the hope that the oxide formed on the surface next to the nerve, would be calculated to soothe any irritation arising after the operation. The great conduction of either of these metals prevented their being successfully employed, and resort was had to other substances. Gums of various kinds were used, such as shellac, copal, mastic, etc. Some ten years ago we were in the habit of using an alcoholic solution of gum mastic, evaporated to great consistency and then thickened to a stiff paste by the addition of plaster of Paris; for a while we thought we had made a hit, but abandoned the use of the article. Asbestos we next employed with very great success, and still make use of it in cases where the roots are filled and we have not at hand the substance of which we have recently made use. Pulverized glass, feldspar, plaster of Paris, Hill's stopping gutta percha alone. We used also cork, raw silk and charcoal, and after numerous trials, limited ourselves to experiment on paper, whalebone, goose-quill, turtle-shell and horn. This last article we have found the best calculated in all respects to fulfill all the requirements. Horn can be pared or filed to any thickness; it may be obtained of any hue. When cut of suitable shape, by immersing it for a few moments in hot water, we have it in an almost plastic state; allowing it to assume such a shape as we may desire, exposure to the atmosphere, or cooling, for a few moments, restores it to its former toughness. We have never been able to manipulate whalebone or goose-quill so satisfactorily, on account of their great elasticity, which would make them fly out of the cavities if pressing upon the sides as is usually required.

We think horn a sufficiently good non-conductor, its indestructibility is well authenticated. When used as a manure for grape vines, in France, horn parings, procured at the blacksmith shop, have been found to retain their integrity after nine or ten years' exposure to the vicissitudes of temperature and the action of the earthy salts.

We have used horn with great advantage in the case of front teeth decayed to a shell; by placing a thin plate of this material against the front enamel, we avoided entirely displaying the color of gold through the enamel, and preserved a natural color in the tooth.

The great similarity and affinity of structure of the substance used as a non-conductor, with that of the tooth, in close contact of which it is of considerable importance in promoting the deposit of bone, said to take place under favorable circumstances, and we know of no substance combining all the desirable requisites to such a degree as horn. We hope the profession will give it a fair and thorough trial, which must result in making horn a valuable addition to our therapeutics.

### A FREAK OF NATURE.

A freak of nature by U * * * * Spruce street, reminds us of a case related by a friend in Mississippi, where the canine tooth had worked its way into the antrum, and produced disease which baffled all remedial treatment, until the floor of the antrum was removed by exfoliation, revealing the true cause of the disease, which was soon relieved after the tooth was removed. We give the case from the News Letter:

A lady, aged, say 50 years, from a neighboring town, presented her mouth for examination, by the advice of two prominent medical men of that region of country, for an enlargement of the palatine surface. Supposing that an indolent tumor, or kindred disease, was developing itself, requiring the application of the surgeon's knife. The lady had several plates fitted to and covering the roof of the mouth; but in consequence of the continued enlargement of this tumor, had not been able to wear them for any length of time. I was induced to ask her to allow me to cut into the enlargement, knowing there was either a tooth or tumor presenting, and suspecting the latter, much to my surprise a beautiful canine tooth presented itself, and on removal was perfect. *Where* this tooth had remained until this period of life remains to be seen, or was it a freak of nature? The peculiar position, 1½ inch from the alveola border, five or six lines to the right of the mesial line, and presenting from within outwards, so that a pair of straight, narrow-beaked forceps was employed for its removal. I have frequently read and heard of a third set of teeth presenting themselves, but this is the nearest approach to that phenomenon that I have ever seen; if the remaining teeth should present in that unruly manner, no doubt the lady will exclaim, "from such, good Lord, deliver us."

### A RECEIPE FOR MOUTH WASH.

The following receipe for a mouth wash, we take from the April number of the News Letter. It is recommended in diseased condition of the gums, resulting from an accumulation of calculi around the necks of the teeth, producing a "conjested and relaxed condition of the capillaries at the margin of the gums," also in ulceration and fungus condition. By D. B. Whipple, M. D., Dentist, Philadelphia.

℞.—Sulphate of alumina, gr. xvi.

Labarraque's solut. chlor. soda ℨij.

Mel. Desp. ℥ss.

Aqua rosa, qt. sft. ℥iv.

Mice and gargle three times a day after meals.

As mistake is liable to occur by the druggist reading the prescription hurriedly, score beneath the *sulphate* of *alumnia,* besides writing it full and plain, lest common alum be substituted, which would change its character materially.

The July number of the American Journal of Dental Science is at hand, and filled with its usual amount of varied and useful matter.

We have first a continuation of chemistry of the metals, by Prof. N. Wright, M. D. The metal under consideration in the present number is mercury, the mode of extracting this metal from other substances is given. Also its combinations with other metals and oxygen. To those who are in the daily habit of plugging teeth with amalgams, this subject would be especially interesting. We may at a future time select such extracts as may be specially important.

ARTICLE SECOND.

In this number, is Dr. Harris' dissertation on diseases of the dental pulp and their treatment. This we are giving our readers entire, and think they will find it worth a careful perusal.

ARTICLE THIRD.

Is a report on the "Rise, Progress and prospects of Mechanical Dentistry." By M. D. French, D.D.S.. The Doctor does not pretend to describe mechanical dentistry, previous to Mr. Bell's time, (say 1829). A correct history up to Bell's time of the dental art, would be difficult to get at. Yet if correctly given, would be instructive and interesting, instructive in showing what has been accomplished and interesting in showing the singular *resorts* by which *beauty* kept up a show of appearance. The article is interesting as giving a history of the progress of this branch of our profession up to the present time; but we have not room for the entire article, and it will not bear much division. As a matter of course nothing specially new is offered.

ARTICLE SIXTH.

This is an article by James Harrison, Dentist on the method employed by him, in counteracting the unfavorable impressions made by chloroform. The remedy is to sicken or nauseate the stomach mechanically. This is accomplished by running the feather end of a goose quill down the throat as far as the larynx, and tickling this and especially the epiglottis. If a quill is not at hand use the finger. Ricord recommends the latter, and Dr. Muzzy of this city has restored two patients in this manner and by artificial respiration.

ARTICLE EIGHTH.

Is cases of caries and exfoliation of the jaw, by Dr. W. A. Shelby. We give the result of one case from this article which had been under treatment by the family physician, about seven weeks, and originated in simple toothache in an inferior molar. It shows how great may be the injury resulting from the presence of a diseased tooth in some peculiar constitutions, and how much pain and suffering might be avoided by timely extraction. We have had two or three cases of like character —one where every tooth in the inferior jaw was lost by timidity of the patient and utter refusal to have some three teeth and their alveolar processes removed at the proper time, notwithstanding we urged this as the only means of saving the remainder of the teeth of that side. His physician (a Homeopathist) insisted he could cure him. In some six weeks after this, we took out three teeth and processes, and urged the removal of all on that side to save the others ; but he again refused, and it thus went on until every tooth was lost. We have always as yet found in these cases, but little hemorrhage, when ulceration and exfoliation had progressed so as to loosen the teeth and processes, not often as much as in ordinary extraction. The Doctor remarks that,

On examing the originally painful tooth, with the intention of removing it as the source of mischief, since it was considerably decayed, I found the soft parts around the tooth destroyed by ulceration, and a portion of the alveolar processes exposed around its roots. This is indicated in the specimen at the present day by a brownish yellow tinge.

In an attempt to shake the tooth with my finger and thumb, I found all the teeth moving simultaneously. Satisfied that a section of the bone was detached by carries and imbedded in purulent matter, I at once determined upon its removal, but not having known the nature of the case, until I arrived at the residence of my patient, I found myself unprepared to proceed that day, so I left and returned the next day, June 2d, "armed," to use the language of the late Dr. Harlan, in his remarks on Prof. Wm. Gibson's operation for osteo-carcomateous tumor of the antrum and superior maxilla, "with horrid crooked knives, gouges, chisels, saws, and even such cruel instruments as red hot pokers," i. e., actual cautery—in short, I prepared myself to meet any emergency with regard to bones and blood vessels. I dreaded, more particularly, the inferior maxillary artery. *A priori*, it was impossible to know in what condition it might be, and a section of it must necessarily be removed with the bone.

I kept my actual cautery in a serviceable condition, and with a small scalpel made a section on each side of the teeth, from the cuspidatus to the last molar. I then grasped the denuded portion of the bone surrounding the roots of the first molar, with a pair of lower molar forceps, and by a vascillating slight traction, brought the entire diseased portion away, with the two bicuspids and two molars in their respective situations. I found much less difficulty in its removal than I had apprehended. There was considerable hemorrhage at first, but it soon yielded to cold, astringents, such as tinct. gallæ in water, and acetat. plumbi in solution. These I ordered to be continued four or five times, daily, and after a few small spiculæ of corroded bone dischared, during the first few days, the discharge almost suddenly ceased, the loose flabby gums contracted and approximated, and new granulations of flesh sprouted from the bottom, closing the wound in a remarkable short time. Every other distressing symptom vanished, her stomach regained its tone and tranquility, and her general system was soon renovated.

### ARTICLE NINTH.

Is by Charles H. Dubs, Dental Surgeon, on improvements in suction plates. This consists in elevating the rugæ of the mouth, or rather elevating the plate which cover the rugae. This allows the plate to rest more equally on the depressed portions, and indeed makes small air chambers wherever the rugæ are "particularly near the crown of the dental arch." This we think was recommended a few years since by some one else, yet we can not now refer to the name. Dr. Dubs takes two impressions, reserving the best to finish the swedging of the plate upon.

We believe that in many cases the plan is a good one, and when executed neatly a narrower plate will be needed than is otherwise used.

### ARTICLE TENTH.

This is on improved sets of artificial teeth, by Mahlon Loomis, Cambridgeport, Mass., and consists "chiefly in making whole or half sets of artificial teeth, all of porcelain, without the use of any metallic plate, and a half set to consist of but one piece or block." The Doctor further remarks, "I know from experience that no dentist will ever go back to other methods after he has once made use of this; and that no person who wears artificial teeth will ever wear any others, when they have tried or seen these." If this be so, Drs. Allen and Hunter had as well abandon their very interesting and protracted suit, for both will soon be forgotten in this new and superior method. It will however take something more than a patent in this country, Great Britain and France, to make dentists swallow all this. Dr. Loomis must beat Drs. Hunter and Allen both, in non-contractability of material, to accomplish all he claims.

ARTICLE ELEVENTH.

Is on a new apparatus for making spiral springs, by C. N. L. Schmedicke, Dentist, Berlin, Prussia. We take it for granted that but few of our readers would like to be seen with a pair of spiral springs; and as we have not the drawings of this apparatus, we can not give the article.

ARTICLE FOURTEENTH.

Gives instructions in the use and management of artificial teeth, by John Tomes, F. R. S., Surgeon Dentist to Middlesex Hospital, London. This article gives us a pretty correct idea of mechanical dentistry in England. In this country it would be called "old fogyism," and yet in Europe we are told many prefer artiificial teeth on ivory base. Mr. Tomes is a good writer and a scientific man. We give an abstract which shows his "usual practice."

It was usual in my practice, and I believe in that of other dentists, to assume the cast of the gums obtained by the present process to be correct, and upon that faith to proceed to construct the teeth to fit the plaster cast, until about four years since, when I had the good fortune to discover means whereby the correctness of the cast could be readily tested. Since this time, I have always availed myself of the test previous to constructing the teeth.

The means I allude to, with other appliances for teeth-making, formed the subject of a patent in 1846. It consists in the compounding of a material like in composition to extremely hard sealing-wax, but which is soft and plastic at the temperature of boiling water, though hard and unyielding at that of the human body. This material, when softened, is molded on the plaster cast into the shape of the required teeth. Thus we have, at a very trifling cost of time, a model of the new teeth, on which, by the aid of a little hot water, we can work any required changes, should it, on being placed in the mouth, need any. And this, of course, will depend on the faithfulness of the cast on which it has been molded. If the cast be correct, the model will fit equally well both the cast and the mouth; but should the cast be faulty, the model made on it will not fit the mouth, whereby we discover the error in the cast, and proceed to its correction. The faulty cast is thrown away, and the composition model is slightly softened by immersion in hot water. When in this state, it is carefully molded to the surface of the gums, and then allowed to harden. When hard, it is again put in the mouth, and if found to fit, is used to furnish a plaster cast in the same manner as the bees-wax mold did in the first instance. By these means we obtain a known *perfect* cast, to which we may make the new teeth without fear of failure. Should gold be chosen for the base, casts in metal, zinc, or brass, are made from the plaster cast, and from these again counter-casts, or reverses in lead are made, between which and the cast, gold plate is hammered, until it has assumed the form, and fits perfectly to the surface of the cast; and, of course, also to the gums.

If dentine be chosen for the base, it is usual to cover the plaster cast with red pigment, and to place upon it a block of dentine in the position it required to take when fitted. The block will at first touch only at, or on two points, and these will be marked by the adhesion of a little of the pigment. The points so indicated are cut away with small tools, similar to those used by engravers. The contact is renewed, and the reddened points again removed. In renewing the contact between the block of dentine and the paint-covered cast, great care should be taken to keep the two in the same relative position as on each preceding occasion. This tedious and somewhat uncertain process is repeated again and again, to the extent of many hundreds of times, till the block is at last cut to fit the surface of the cast. The superfluous portions are then removed, and the base so made is prepared for the reception of the teeth.

In my own practice, the base is carved by a patent machine, which altogether supersedes the hand carving and use of pigment. A model of the required teeth is made in the molding composition, and this is fixed in the machine, and then copied into dentine with much saving of time, and without liability to error.

For the invention of this instrument, I had the honor to receive a gold medal from the Society of Arts.

There are, however, a few workmen to be met with, who, from great practice and a considerable amount of ingenuity, produce results that can scarcely be surpassed. But such men are not numerous; hence, their services can not at all times be commanded.

The base, so far as its gum-fitting surface is concerned, having been finished, we have next to select the teeth which the base is destined to carry.

Teeth used in making artificial teeth are of three kinds: human teeth, mineral and carved teeth—that is, teeth carved out of dentine. The latter, when dentine is used for the base, are carved out of the same block in one piece. When natural or mineral teeth are selected, they are fixed to the base by pins.

In speaking of artificial teeth, dentists divide them into front teeth and side blocks. The front teeth are like, and have the same names as the natural teeth, including the bicuspids; while those corresponding to the molar teeth are made in one continuous piece, and are called the side-blocks of the piece. I should here tell you that the teeth, whether few or many, with their base, when spoken of as a whole, are termed by dentists a *piece*—an upper or under piece, as they may be for the upper or lower jaw.

The teeth are fixed to the base by pins passing through, or nearly through the center of each tooth, and soldered to the gold, or riveted through the dentine, according as the base may be composed of the one material or the other.

Should a tooth, when in wear, come off its pin, it may be temporarily refixed by wrapping a little fine silk round the pin, and then replacing the tooth.

### ABSORPTION OF DENTINE.

We have in the April and also July number of the News Letter, an article on this subject by J. D. White, M. D., D. D. S., which are interesting, and worth careful perusal. If these are fair specimens of the Doctor's *note-book*, we should think if it was all published it would make a neat and valuable volume. We give from the July number, case No. 1, illustrative of absorption of the dentine in the crown of the tooth, from the action of the vessels of the pulp cavity:

A young gentleman, 20 years of age, and of a nervo-lymphatic temperament, was presented to us by a distinguished neighboring dentist for consultation. It had been observed by his dentist, that a faint red spot was making its appearance in one of the front teeth, opposite the apex of the pulp; and which was the cause of some solicitude on the part of the patient, as well as the dentist. Of course it was a question, what could give rise to this strange appearance. We remarked at once, that it was one of two things—either a small blood vessel had been broken, and the dentine was absorbing the red globules of the blood, or it was a fungus of the pulp, which was displacing the dentine in some way; and this fungus was highly vascular, and was filling up the excavation as rapidly as it was made; and that it would go on, in all probability, until it would carry away all the internal structure of the tooth. We gave directions that it should be watched, and by taking the exact size of the spot at the time, with a compass, and compare it with its size at a given future period, it could be determined whether it was increased, and at what rate, etc. This precaution, however, was not taken, and in about one year the patient called upon us, and requested us to do something for the case as soon as possible, as the tooth had now become of quite a pink hue. The coloring matter had evidently approached to the anterior and posterior plates of the enamel, but the enamel appeared much thinner on the posterior part of the tooth. We proposed at once, to pass a drill through the enamel at the back part of the tooth, as there was no cavity of decay about the tooth by which the spot could be reached, but we had scarcely given our drill more than five or six turns, when it plunged into a large cavity, filled with a vascular mass of very low sensibility. After the bleeding subsided, we placed in contact with it the usual arsenical paste for treating the nervous pulp ; and in about twenty hours after, we scraped away a large amount of the mass ; but on going down the root, we found the sensibility to increase, as we approached the normal structure of the pulp. We applied a small portion of the paste a second time, and in about twenty hours after removed the pulp, about two-thirds or three-fourths of the length of the root. The inner surface of this absorbed cavity was rough, and the substance of the tumor or fungus was adherent to it.

The absorbing process does really seem to attack the enamel also, as we have specimens of this kind in our possession, but it does not attack a decayed spot on a portion of the tooth, which has undergone such a change as to have lost its vitality, as this absorption can be observed to stop; when it meets with a cavity of decay from the outside, it works around it, leaving the decayed portion stand free, as though the decay had been cut away from the sound tooth. This may be when it allows the enamel to break in, or exposes a plug that has been put in a cavity with only a thin plate of dentine to protect it ; but not so, it is a *vital act*, while what we familiarly know as decay is *chemical, solution* by contact with external agents. How else could we explain the fact, that many teeth lose their enamels, and the sensitive and vital bone still for a long time continues to resist the destroying agents.

The tooth above described, was washed out daily with water, so as to prevent the blood from remaining in it for a length of time, and in ten days the whole cavity was firmly plugged, as an ordinary case of treatment of the pulp. Three years have elapsed, and it still remains unchanged. On one occasion it gave some pain, which was relieved by the application of a few leeches to the gum.

### CASE OF HEMORRHAGE.

The July number of the News Letter contains an interesting case of hemorrhage, which was relieved by pressure, after resisting the use of astringents, styptics, etc., by J. F. Leaming, Seaville, N. J. With Dr. Leaming we believe that styptics only "aggravate the evil by exciting increased capillary irritation, or by forming a slough, which will certainly reproduce the hemorrhage." We have found that each application of a styptic only increased the bleeding surface. But in two or three cases in adults, pressure failed to arrest, and it was only by constitutional treatment that hemorrhage was finally arrested. The contrivance of Dr. Leaming for producing pressure is simple and ingenious, and we give it:

Turning my attention fully to the only available remedy remaining, *compression*, and feeling that its full effect had not been obtained in consequence of the restlessness of the child, and the impossibility of making uniform pressure with the finger, I contrived a spring out of a few narrow strips of tinned sheet-iron, with a compress at one end, as nearly as possible adapted to the gum, and sprinkled with powdered gum arabic; the other end being bent around so as to rest under the symphasis mentis. This being carefully padded, was adjusted so as to give the full force of the spring upon the bleeding gum, and retained in its position by a suitable bandage.

After forty-eight hours the compress was removed, having arrested the hemorrhage perfectly. During the above treatment the acetate of lead and anodynes were freely used. The child eventually recovered.

This child was of *the hemorrhagic diathesis,* as I have since ascertained by serious hemorrhage resulting from every slight wound, yet nothing in the parents or other members of the family would lead to that conclusion; so that *inquiry* could not have prevented the accident.

### NEW METHOD OF MAKING BLOCK TEETH.

We have also in the same number of the News Letter a "new method of making block teeth." This to many of our readers who are prepared for that kind of work, would be interesting. The article to be properly understood would have to be given entire, and we have not room for it in this number. We may refer to it again.

### "AUDI ALTEREM PARTEM."

We have next a review of "*Audi Alterem Partem*," by Dr. A. S. Talbert, of Lexington, Ky. We are glad to see our friend Talbert has been drawn from his retirement, and hope that one who is capable of writing so well will more frequently let his name appear in our periodicals.

### FILING TEETH TO RELIEVE PAIN PRODUCED BY PRESSURE.

The July number of the News Letter contains an article on "filing teeth to relieve pain produced by pressure in their development," by W. H. Baker, M. D. As a remedial agent we are fully convinced of its utility, but as a means of saving the teeth how much better it would be to extract one of the teeth—say the third molar.

In all cases where the molar teeth are filed, we have found it necessary to make large spaces with good shoulders to prevent the filed surfaces from coming in contact. Unless this is done decay and ultimate trouble will ensue. We give an abstract embracing the Doctor's views:

If the third molar is distorted in its position, or is such in growth that there can be little hope of its becoming an efficient organ—the duties of which, if it does not fulfill—its presence must prove worse than useless, and therefore it should be removed. But, as not unfrequently happens, the patient's fears, aided by the consciousness of the distant seat of pain, frustrate the surgeon's judgment; it is then that I would urge its pressure against the next being removed by a file being passed between it and the second molar, and this part of the operation to be performed previously to that of a file being passed between those teeth which occupy the seat of pain, and not unfrequently it will be found to preclude the necessity for the latter being done, and if so, the most valuable are preserved uninjured.

Another test by which the truthfulness of the existence of abnormal pressure may be diagnosed, is to be found in the increased pain being given, or its reproduction from cessation caused, by the introduction of a thin wedge-shaped instrument between either the teeth affected, or between the second and third molar. This instrument increases the pressure, and therefore, increases the pain, and the true origin of the disease is palpably manifest.

Generally the operation of the file, in such cases, is described as far from producing so disagreeable a sensation as under ordinary circumstances, and the completion of the operation is always instant relief. Sometimes, if the enamel be thin, or the approximal surfaces lie very parallel to each other, so that the file would have to cut its whole way to the gums, coming into contact with the dentine and peri-dental membrane at the extremity of the enamel, or from some other cause the tooth may be exceedingly sensitive, the operation which would otherwise be very tedious and painful, may be greatly relieved by the local application either of chloroform, or a strongly saturated solution of camphor in rectified spirits of wine. Of course, as in all long operations of the file, the frequent application of cold water, by precluding the file and tooth from becoming hot by friction, renders the operation safer and more pleasant to both the operator and patient.

DENTITION—ITS CONNECTION WITH IRREGULARITY.

The July number of the News Letter contains an article on this subject, by Robey Augustine, urging very properly the retention of the deciduous teeth until the teeth of replacement are ready to take their place. Recommending also the study of "Carpenter's Physiology of Dentition." We would also urge on the young members of the profession more attention to this subject. We see almost every day evidences of the injury resulting from the too early extraction of deciduous teeth.

IRREGULARITIES OF TEETH.

The same number of the News Letter contains an article on this subject, by H. S. Burr, M. D., giving the drawings of a case, and mode of treatment. The treatment is much the same as that pursued by ourself, only we do not use the skeleton cap to the molar teeth. We cap the teeth that antagonize with the teeth striking inside of the under teeth, and with an incline plane make the proper articulation; then use the bar and patent thread ligatures. We are glad to see by our Dental periodicals that more attention is of late given to this subject.

THE PRACTICE OF DENTISTRY IN PORTUGAL.

In the July number of the News Letter, we have a letter to the editor, from W. C. Starbuck, jr., Lisbon, Portugal, giving us some idea of the practice of dentistry in that country, and the trouble of getting a certificate or permit to practice. It appears that an American diploma would have saved Mr. Starbuck much trouble and expense. We think gentlemen leaving this country for the practice of dentistry, should always take with them a diploma from some of our schools. They may rest assured that if not regarded at home as of any consequence, they will be considered abroad as of some value. But we are satisfied that both at home and abroad they are regarded as the best certificate of merit that can be obtained. As an evidence of this we would state that at our last commencement, or during the last session of our school, we had letters from more points, requesting us to send a graduate of our school to settle permanently with them, then we had graduates to send. We could have supplied twice as many locations as we had graduates—and they were all good locations, some of the best in the country—yielding a practice of from one to five thousand dollars per year. We give an abstract from the letter of Mr. Starbuck:

It is only necessary for me to mention one incident in proof of my assertion. The Portuguese dentists are in the habit of using lead or an amalgam for plugging teeth; and, a few days ago, one of them called on me, requesting me, as a great favor, that I would give him a little amalgam to plug a tooth with, as the patient was in a hurry, and he had no time to prepare any. I told him that I used gold only, at the same time showing him some specimens of plugging. He stood transfixed with astonishment, and after many exclamations of admiration, asked me, "*if I poured the gold in hot.*"

I trust that if there is any young dentist who thinks of visiting a foreign land, or even settling at home, and who has not received the highest honor which can be conferred on him by one of the Dental colleges, which are to be found in no country but ours, that he will take warning by my experience and misfortune, and lose no time until he has obtained that best of all recommendations, *a collegiate attest.*

### AUROPLASTIC BASE FOR ARTIFICIAL TEETH.

The July number of the Dental Recorder, has an article on this subject by the editor Dr. Hill, which is rather a continuation of former articles on this subject. Dr. Hill it is known has been making experiments in the use of gutta percha and particularly "*Hill's Stopping.*" This substance is colored to imitate the gums.

Experiments so far in the hands of Dr. Hill appear to increase his faith in the utility of this article. As yet, however, it is only recommended for temporary purposes. The modus operandi is thus described by the Doctor:

In the use of this material, the first thing to be done is to secure a good plaster model of the mouth. Of course it should be as near perfect as possible. When this plaster model is dry, take a small pencil brush and paint over its surface with good olive oil, to prevent the plaster from adhering to the compound. This is important, and oil is much better than varnish for this purpose. This done, the next thing is to cut a pattern for your plate, just as if you were going to use gold or any other material. It may be of sheet lead, zinc or paper. Next take "Hill's Stopping," *rolled smoothly down to a thin sheet.* It may be nearly as thin as heavy gold plate, especially if it be desirable to make the piece very light. This may be accomplished as follows: Get a smooth marble slab, say 10 or 12 inches in length, by 8 inches in width, and an ordinary rolling pin, such as women use for pie-crust. Now dip the compound into clean hot water, lay it upon the marble slab, and roll it out as thin as you desire. By the use of *hot water* the compound may be softened at pleasure. Having your compound rolled out thin as may be desired, lay your pattern upon it, mark it with a pencil, and cut it out with ordinary scissors. It is now ready to be moulded to your plaster model.

For this purpose have a vessel of clean hot water, dip the compound into it and let it soften, then carefully lay it over the model and with the thumb and finger press it down firmly until it becomes a perfect fit. When this is done, take a sheet of *fine wire cloth,* such as is used for making sieves or strainers, either iron, steel or brass, (the iron is least objectionable)—place your *pattern* over it, mark it out a little smaller than the patern, so that the edges may all be covered up, and cut it out in the same way that you would cut out the original base.

Then bend it over the model, shaping it as you like ; when it is shaped to your liking, heat it, and press it firmly upon the base, until every part is held by that material and imbedded in it. This done, cut a covering for it precisely in the same manner as before out of the rolled compound, dip it into the hot water and lay over the whole, pressing firmly down as before.

If this has been carefully attended to you will now have a neat, substantial base, ready to be tried in the mouth. Any alteration now can be easily effected. The edges may be trimmed with a pen knife, or in any way added to or diminished, as circumstances require.

If it be found to fit the mouth accurately you may then proceed as follows : Replace it upon the plaster model, and cover the alveolar ridge with an extra thickness of the compound, warmed, and pressed firmly upon it. It is now ready for mounting. The next thing is to mark the centre line upon the model. This done, we proceed as follows : Having selected our front teeth, we seat ourself at the bench, with the model before us and a small spirit lamp burning at our right hand. We now seize the front incisor tooth with a pair of small plyers and hold it for a moment in the blaze of the lamp, and then press it into the compound in its proper position. If heated to the right temperature this is easily and quickly accomplished. When the front incisors are in place, we try it in the mouth again to see that they are right. If any alteration is needed it is now made. This done, we proceed with the other teeth in the same way, occasionally trying them in the mouth and antagonizing them as we desire, until the teeth are all in place. And now, all that remains, is to confirm the teeth in their places, and finish the piece. For this purpose, let them still be retained upon the model. Then take a small strip of the gum-colored compound of any desired thickness, lay it upon a clean tin sheet and hold it over the blaze of the spirit lamp until it is sufficiently heated, and then lay it all around the base of the teeth, front and back, massing it down with a *smooth flat-pointed* instrument, heated in the blaze of your spirit lamp. A little practice will soon suggest the kind of instrument best adapted to this purpose. And thus, by occasionally heating your instrument, you may build and shape the gums in any way your taste may dictate. And by a slight admixture of various shades of the compound, the gums may be painted to look very natural and pretty in the mouth. Two or three points in this process should be carefully observed :

1st. When a *dry* heat is used to soften the material, whether it be on a plate of tin or porcelain or other substance great care should be taken *not to burn it*, as this would destroy its strength and usefulness.

2d. If *iron* or *brass wire cloth* is used, *every portion should be entirely covered by the compound*, so that the fluids of the mouth will not act upon it. Silver or gold wire cloth would be preferable, but these, perhaps, would be somewhat difficult to obtain at present.

3d. If any change in the position of the teeth is required, after they are set, it can easily be effected by holding the points of the teeth in the blaze of the spirit lamp, until they become heated, and communicate their heat to the material that surrounds them. At this juncture they can easily be adjusted.

4th. *Pivot teeth* will generally answer the best purpose for this kind of setting. And these we prefer for the upper fronts. For *under sets*

Jones, White & Co.'s continuous gums are the best. These are ex ceedingly neat and pretty, when mounted, as they can be, with this material. There is not the slightest difficulty in making under sets of great strength and usefulness of this kind of work. We have thus endeavored to set forth in a plain and intelligible manner, our method of using the "auroplastic base" for artificial teeth. One or two observations will conclude what we have to say upon this subject for the present.

Although we hold a *patent* for this compound, and this patent covers its use in any form, we shall allow any and every one to use it who may see fit to do so *only reserving to ourself the privilege of making and vending the compound thus used.*

If any dentist desires personal instruction in the use of this material, (and more can be learned by seeing the thing done, in fifteen or twenty minutes, than in reading a volume upon the subject), we will cheerfully show them, for a moderate compensation, if they will visit us for this purpose, charging them only for the tax upon our time.

The compound if properly used, will not be found very expensive, as it is quite bulky, and an ounce will go farther than is ordinarily supposed. We have thus taken pains to anticipate any question which would naturally suggest itself, and trust our readers will obtain hereby all necessary information in regard to "auroplastic base."

We publish the resolutions offering a prize of one hundred dollars for the best Essay on Dental Surgery, to be handed in by the first of January next. We hope the Profession will think of this, and send in by that time something which the committee will take pleasure in presenting at our next meeting.

In relation to the *indefiniteness* of the resolution as expressed by the phrase "Dental Surgery;" we would remark that the design we believe of the Society, was to leave some latitude to writers, wishing, however, to illicit the best Essay for general distribution among our patients and the Profession. That which conveys the most and best practical and scientific matter in the given space, we presume will stand the best chance for the prize.—[Ed.

*Resolved,* That this Association will award one hundred dollars to the Author of the best Essay, (not previously published), on Dental Surgery, *adapted to popular circulation,* to contain not less than fifty nor more than seventy-five pages duodecimo ; the copyright to be the property of the Society. Said Essay to be approved by a Committee, and by it to be printed and issued to the members of the Society, and receive the approval of the Association before it be published for general circulation.

*Resolved,* That a Committee of five be appointed by ballot, to examine the Essays presented, and report at the next meeting.

Essays competing for said award to be forwarded to the Chairman of the Committee as early as January 1st, 1855.

Election of said Committee was had, and the following gentlemen appointed: JAS. TAYLOR, W. H. GODDARD, A. BERRY, A. M. LESLIE, and J. TAFT.

N. B. Authors sending Essays under the above resolutions, will accompany it with their full signature and address, which they will enclose in a sealed envelope, which will be opened should the accompanying Essay prove the successful one; otherwise, it will be returned with the manuscript.

GEO. WATT, Cor. Sec'y M. V. A. D. S.

